# Dining out: developing an outreach service for a busy eating disorders program

**DOI:** 10.1186/2050-2974-1-S1-O18

**Published:** 2013-11-14

**Authors:** Edward Mullen, Lisa Stokes

**Affiliations:** 1 Eating Disorders Unit, NorthWestern Mental Health, Royal Melbourne Hospital, Australia

## 

The Eating Disorders Program (EDP) based at Royal Melbourne Hospital offers inpatient, day patient and outpatient services to adults from western Melbourne and regional Victoria and receives over 150 referrals per year. Despite streamlined referral processes to the program and access to training for front line clinicians, there are still gaps in the management of eating disorders in the community. Most affected are patients who have been discharged from the inpatient or day programs and those from regional centres.

The EDP set up a pilot outreach service in 2013 following extensive service mapping and needs analysis. There were two initial goals; individualised community treatment for patients in recovery phase of their eating disorder and a consultation liaison role to advise local and regional clinicians and inpatient units in effective management of eating disorders.

The outreach service consisted of a multi-disciplinary team supported by the EDP. The goals of treatment for individual work were improved quality of life, weight restoration and decreased eating disordered behaviour. The consultation liaison role aimed to reduce the need for admission to specialist beds and build the capacity of local clinicians.

This presentation aims to describe the background, setup and outcomes of this new service.

This abstract was presented in the **Care in Inpatient and Community Settings** stream of the 2013 ANZAED Conference.

